# Objectifying Specific and Nonspecific Effects of Acupuncture: A Double-Blinded Randomised Trial in Osteoarthritis of the Knee

**DOI:** 10.1155/2013/427265

**Published:** 2013-01-10

**Authors:** Max Karner, Frank Brazkiewicz, Andrew Remppis, Joachim Fischer, Oliver Gerlach, Wolfgang Stremmel, Shanmuga Velayutham Subramanian, Henry Johannes Greten

**Affiliations:** ^1^Department of Internal Medicine, Heidelberg University Hospital, Im Neuenheimer Feld 410, 69120 Heidelberg, Germany; ^2^Centre for Chinese Medicine, Universitätsallee 3, 28359 Bremen, Germany; ^3^German Society for Traditional Chinese Medicine (DGTCM), Heidelberg University, Karlsruher Straße 12, 69126 Heidelberg, Germany; ^4^Institute of Public Health, Social and Preventive Medicine, Mannheim Medical Faculty, Heidelberg University, Ludolf-Krehl-Straße 7-11, 68167 Mannheim, Germany; ^5^Shen-Centre for Traditional Chinese Medicine, Südliche Stadtmauer Straße 25, 91054 Erlangen, Germany; ^6^Department of Society, Human Development and Health, Harvard School of Public Health, 677 Huntington Avenue, Boston, MA 02115, USA; ^7^Abel Salazar Biomedical Sciences Institute, University of Porto, Rua de Jorge Viterbo Ferreira No. 228, 4050-313 Porto, Portugal

## Abstract

*Introduction*. Acupuncture was recently shown to be effective in the treatment of knee osteoarthritis. However, controversy persists whether the observed effects are specific to acupuncture or merely nonspecific consequences of needling. Therefore, the objective of this study is to determine the efficacy of different acupuncture treatment modalities. *Materials and Methods*. We compared between three different forms of acupuncture in a prospective randomised trial with a novel double-blinded study design. One-hundred and sixteen patients aged from 35 to 82 with osteoarthritis of the knee were enrolled in three study centres. Interventions were individualised classical/ modern semistandardised acupuncture and non-specific needling. Blinded outcome assessment comprised knee flexibility and changes in pain according to the WOMAC score. *Results and Discussion*. Improvement in knee flexibility was significantly higher after classical Chinese acupuncture (10.3 degrees; 95% CI 8.9 to 11.7) as compared to modern acupuncture (4.7 degrees; 3.6 to 5.8). All methods achieved pain relief, with a patient response rate of 48 percent for non-specific needling, 64 percent for modern acupuncture, and 73 percent for classical acupuncture. *Conclusion*. This trial establishes a novel study design enabling double blinding in acupuncture studies. The data suggest a specific effect of acupuncture in knee mobility and both non-specific *and* specific effects of needling in pain relief.

## 1. Introduction 

Knee osteoarthritis is a major cause of morbidity, disability, and health care utilisation, particularly in elderly patients [1]. The primary clinical manifestations are pain and joint stiffness [2]. Therapy recommendations aim to improve physical function and to relieve symptoms [3]. Unfortunately, pharmacological approaches often render limited effects and also carry the burden of potentially serious side effects [4]. Hence, many patients try complementary medicine treatments [5–7]. Amongst the nonpharmacological approaches, the use of acupuncture has increased consistently during the past few decades [7]. 

Recent randomised controlled trials have produced rather contradictory results with respect to acupuncture's effects. Some trials have suggested a potential benefit of acupuncture beyond that of sham or minimal acupuncture [8–10], whereas other studies have reached the opposite conclusion [11]. These inconsistent results have generated much discussion in the scientific community as to whether the effects in acupuncture were caused by mere skin penetration or by the stimulation of specific points [12–16]. In an attempt to clarify this issue, we found corresponding inconsistencies in the study designs themselves: the sham or minimal acupuncture procedures used as controls in the aforementioned trials differed systematically from the actual acupuncture groups regarding number, size and length of needles, and intensity and duration of the doctor-patient encounter. Moreover, the trials failed to achieve complete blinding [8–12]. Any attempt to clarify the issue of efficacy in acupuncture requires a more controlled study design. 

The controversy over acupuncture extends to the issue of the most effective method of acupuncture [17]. Some practitioners favour a *modern acupuncture*, treating patients according to a semistandardised set of disease-specific points. Other practitioners adhere to an individualised *classical acupuncture,* which derives acupuncture points from an assessment of disease modalities and a physical examination, including Chinese tongue and pulse diagnosis and the localisation of paraesthetic pressure points [18, 19]. 

To elucidate these open questions, we conducted a repeated measures, double-blinded, and placebo-controlled, multicentre trial in patients with chronic osteoarthritis of the knee. The study compared the effects of three modalities of acupuncture (sham, semistandardised modern and individualised classical) within two parameters: joint mobility and pain [20, 21].

## 2. Materials and Methods

### 2.1. Patient Population

Patients aged 35 years or older were recruited by newspaper advertisements and from the outpatient clinics of the three participating centres. Potential participants were first screened by telephone interview, followed by a clinical examination to ascertain the satisfaction of the diagnostic criteria of the American College of Rheumatology and the presence of a severity grade of II or III according to the radiological Kellgren classification. Patients with congenital or traumatic deformations of the knee, malignant disease, autoimmune disorders, surgery or arthroscopy during the past 12 months, medication with steroids, physical therapy, or acupuncture within the last four weeks, as well as intake of opioids during the study period, were excluded from the study. Patients were allowed to continue their regular medication including NSAID or COX2-inhibitors while participating in the study, but changes in medication and dosage were not allowed. The local ethics committee approved the protocol. All patients provided written informed consent.

### 2.2. Intervention, Randomisation, and Double Blinding

Patients were informed that the study aimed to identify the most effective of three acupuncture techniques, including one sham technique. Participants were allocated in random order to (a) the needling of non-specific points (*sham*), (b) a semistandardised selection of disease-specific acupuncture points as used in recent studies (*modern acupuncture*), and (c) an individualised selection of acupuncture points determined by the diagnosis according to the traditional Chinese medicine (*classical acupuncture*). Each patient received all three forms of acupuncture (a, b, and c) in a random order. Each session was spaced seven days apart resulting in one single treatment per week as well as one single treatment per form of acupuncture (a, b, and c). Prior to every acupuncture session, a fully qualified and experienced physician and acupuncturist established the Chinese medical diagnosis as defined by the Heidelberg Model of Chinese medicine [22]. Using three differently coloured pens at random choice, the first physician marked points for *classical, modern, *and* sham acupuncture*. Thereafter, the first physician informed the study-coordinating centre about the colour allocation. The study-coordinating centre compared these colour codings to the sequence of treatment modalities according to a computer-generated randomisation table and informed a second physician about the colour of the points to be needled. In all study centres, this second physician was a novice to acupuncture in order to minimise possible biases arising from the observation of points. This apprentice practitioner was instructed to maintain a standardised method as to needle insertion or needle stimulation throughout all three sessions. After acupuncture, the patients redressed with light garment to cover any potential marks from needling. Thereafter, the patient returned to the first physician, who was unaware of the used acupuncture method, for assessment of pain and knee flexibility. 

### 2.3. Acupuncture Technique

Acupuncture was performed using 0.22 × 40 mm copper needles. Only one knee was treated in the study. Ear and hand acupuncture was not allowed. During all sessions, the number of needles, the type of needles, the depth of insertion, and the intensity of stimulation were kept identical. In each session, ten points ± two points were allowed to be stimulated. The needles were rotated immediately after insertion and again after 15 minutes. Needles were then withdrawn after 30 minutes. Communication with the patient during the acupuncture procedure was minimised to an explanation of the procedure. 

The only systematic difference across the treatment modalities was the location of needling points. *Modern acupuncture* adhered to previously recommended methods for selection of points for knee pain (ST36, ST34, EX32 twice, SP9, SP10, SP6, GB34, LI 4) [11, 23]. In addition, up to three further points were admissible (e.g., *ashi*, LI3, ST40). Non-specific needling used the points described in Table 1. The points for the *classical acupuncture* were determined individually for each patient according to the classical Chinese diagnosis, which assessed the modality of symptoms, complaints associated with certain movements, tissue tenderness along the postulated acupuncture channels, tongue diagnosis, and pulse quality. In contrast to the *modern acupuncture* treatment, the *classical acupuncture* resulted in a larger variation of needling points between patients with a certain overlap to the points selected in *modern acupuncture*. (Data were not shown; statistics on the selected points are available from the authors.)

### 2.4. Outcome Measures

Reasoning that pain-related restrictions in knee flexibility are more direct external measure of pain than subjective self-reported measures, we a priori defined knee flexibility as the primary outcome measures and the WOMAC scale as the secondary outcome parameter [24, 25]. Knee flexion was measured in standardised fashion by using a universal goniometer, aligned with the greater trochanter, through the lateral joint line to the lateral malleolus. The first physician bent the knee to the point at which pain limited further flexion. Knee flexibility was measured before acupuncture, immediately thereafter, and after 7 days (for session two and three, the latter coincided with the baseline-measurement prior to the next treatment). The abbreviated WOMAC pain score was determined prior to acupuncture and immediately thereafter, as well as three and seven days after treatment. Change scores for either outcome were calculated by subtraction of post- from preacupuncture measurements, with a positive change score indicating improvement. For dichotomous outcomes, a treatment success was defined as an improvement of the knee flexibility by 10 degrees or more or a reduction of the WOMAC pain score by 50 percent or more, respectively. 

### 2.5. Statistical Analysis

Knee flexibility as the primary outcome parameter served to determine the sample size. An improvement by 10 degrees was regarded as potentially clinically relevant, and a difference of 5 degrees was viewed as a marginal difference. Based on a pilot study, we estimated a required total of 100 patients to obtain a power of 90% at a type I error of less than 5% in order to demonstrate a difference between methods in knee flexibility change scores by 5 degrees (StateMate 2, Graphpad Software Inc., San Diego, CA, USA). We aimed to recruit 125 patients to allow for dropout and noncompliance. Knee flexibility was shown to be a reliable and valid parameter in several studies [26–29].

Fisher's exact test or the Kruskal-Wallis test was employed to compare baseline characteristics of the three groups resulting from the first randomisation. The main analysis comprised a two-factor analysis of variance (treatment modality and time) with repeated measures. Least square means, 95% confidence intervals for knee flexion, and WOMAC scores were estimated for each patient while taking into account the covariates of gender, premedication (yes or no), disease severity (Kellgren II versus Kellgren III), and number of needles applied. Within subject contrasts were adjusted using the Greenhouse-Geisser correction. 

Repeated measures analysis of variance does not readily provide for explicit modelling of possible carry-over effects. We expected that the effect size of the intervention in weeks 2 or 3 might depend on the treatment of the preceding week. Therefore, we employed multilevel, hierarchical, random-intercept, and random-slope modelling of the change scores in knee flexion. In these models, we nested the six change scores (immediately after the treatment and 7 days after the treatment for all three modalities) within patients. The order of treatment and the preceding treatment were entered as dummy variables. All possible interactions with the treatment modality were systematically explored with non-specific needling and the first treatment as the respective reference categories. Particular attention was given to the modelling of carry-over effects from *classical acupuncture* to *modern acupuncture* and vice versa. In a final step, we explored random intercepts/random slopes of the fixed effects model, as long as the −2 log-likelihood value significantly improved [30, 31]. Blinding was maintained during the statistical analysis.

All analyses were on an intention-to-treat basis. Analyses of variance were conducted using SPSS version 12 (SPSS Inc., Chicago, IL, USA), multilevel modelling employing MLwiN (Version 2.02, Multilevel Models Project, Institute of Education, London, UK).

## 3. Results and Discussion

**Figure 1:**
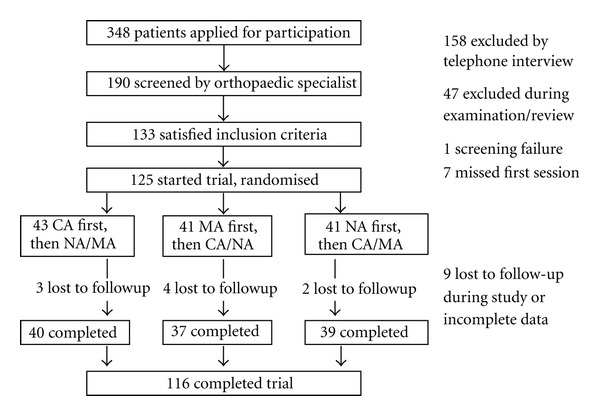
Patient recruitment, randomisation and followup.

One-hundred and sixteen patients (mean age 62.4 years, range = 40–83, 33% males) with chronic osteoarthritis of the knee completed the study between April 2004 and May 2005.  Figure 1 displays the patient recruitment, allocation, losses to followup, and exclusions. Randomisation resulted in a similar distribution of gender, premedication, and disease severity across the allocation for the first treatment modality (Table 2).

**Figure 2:**
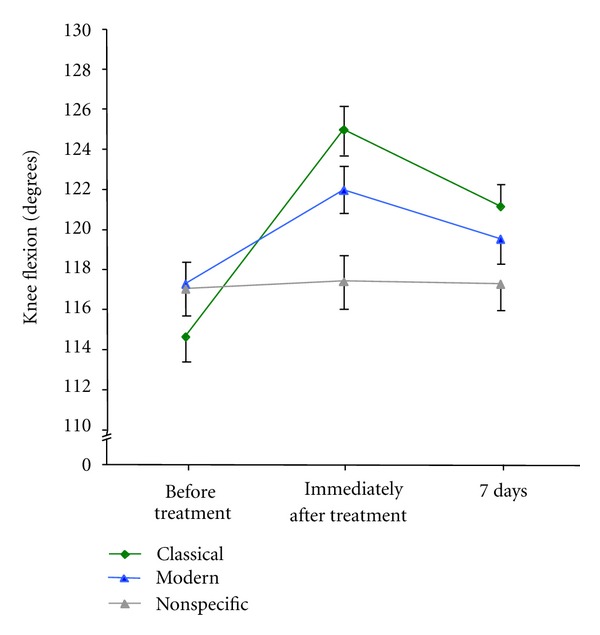
Knee flexion before and after acupuncture. The figure compares the maximum possible knee movement until further flexion was blocked by pain for classical acupuncture, semistandardised modern acupuncture, and non-specific needling. Flexion was assessed immediately prior to treatment, directly thereafter and at a recall visit after 7 days. Data display the means adjusted for Kellgren classification, prior intake of medication, and patient gender. Error bars indicate the standard error of the mean. Knee flexion is displayed in degrees according to the neutral-zero method.

Knee flexibility improved by 10 degrees or more immediately after the acupuncture procedure in 75 of 116 *classical acupuncture* sessions, giving rise to a number needed to treat (NNT) of 1.5 (95% confidence interval 1.4 to 1.8); this compared to 41 of 116 *modern acupuncture* sessions (NNT = 2.9, 95% CI 2.2 to 3.8) and to 6 of 116 non-specific needling sessions (NNT = 19, 95% CI 9.2 to 53, *P* < 0.001). *Classical acupuncture *resulted in a significantly larger improvement immediately after the treatment (Figure 2, mean change = 10.3 degrees, 95% CI 8.9 to 12) compared to *modern acupuncture* (4.7 degrees, 95% CI 3.6 to 5.8), while no effect was observed for non-specific needling (0.34 degrees, 95% CI—0.61 to 1.3; *F* = 27.3; *df* = 3.1, 358; *P* < 0.001). Adjusting for the Kellgren classification revealed that the difference between *classical acupuncture* and *modern acupuncture* was even larger in patients with more severe illness (*P* = 0.02).

The analysis of the change scores employing multilevel modelling revealed significant carry-over effects from the first to the second and from the second to the third treatment. When the first treatment consisted of *classical acupuncture* (estimated mean change = 9.1 degrees, 95% CI 6.2 to 13), the effects from *modern acupuncture* (mean change = 0.7 degrees, 95% CI—1.3 to 2.7) were negligible. However, when the first treatment consisted of *modern acupuncture* (mean change = 5.5 degrees, 95% CI 3.1 to 7.9), subsequent *classical acupuncture* resulted in a further flexibility gain (mean change = 4.3 degrees, 95% CI 2.0 to 6.6). The small differences from the values reported in the preceding paragraph arise from the adjustment for carry-over effects. 

The multilevel model also suggests that the substantial variation between patients in the effects of *classical acupuncture* is relatively independent of the variation in the effect of *modern acupuncture*—in other words, the extent of improvement after classical acupuncture is not correlated with the extent of improvement after modern acupuncture (*P* = 0.43 for the covariance in the random part of the model). 

**Figure 3:**
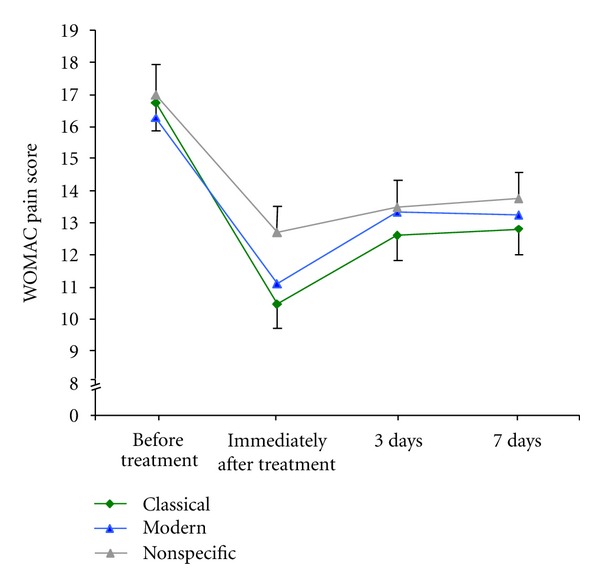
WOMAC pain scores before and after acupuncture. The figure compares the WOMAC pain scores for classical acupuncture, semistandardised modern acupuncture, and non-specific needling. Pain was assessed immediately prior to acupuncture, directly thereafter, by self-administered questionnaire at home at 3 days after acupuncture, and at a recall visit after 7 days. Data display the means adjusted for Kellgren classification, prior intake of medication, and patient gender. Error bars indicate the standard error of the mean.

In contrast to the differences in efficacy for knee mobility, all three treatment forms resulted in some immediate improvement of pain scores (Figure 3). *Classical acupuncture* showed a significantly larger improvement immediately after treatment than non-specific needling did (post-hoc contrast, *F* = 5.4, *df* = 1, *P* = 0.022). Success rates defined as a WOMAC reduction by 50% were the largest immediately after *classical acupuncture* (85 of 116, NNT 1.4, 95% CI 1.23–1.56) as compared to *modern acupuncture* (74 of 116, NNT 1.56, 95% CI 1.38 to 1.84, nonsignificant difference) and non-specific needling (56 of 116, NNT 2.1, 95% CI 1.68–2.46, *P* = 0.02). The pain relieving effect of any needling rapidly declined. At the 7-day follow-up visit, pain scores were similar across the three methods (Figure 3).

### 3.1. Strengths and Weaknesses

The strength of the present study is its use of a novel study design for acupuncture which establishes blinding of both patients and the treating physicians. This design overcame major shortcomings of previous studies which failed to achieve adequate blinding and in which sham treatment usually differed substantially from acupuncture. The results of the present study offer an answer to the basic question of whether the effects in acupuncture are specific or caused by mere skin penetration. In our study, the needle location remained the only difference between the three treatment modalities, approximating for the first time the principles of randomised and double-blinded, controlled trials in acupuncture studies. 

116 patients with osteoarthritis of the knee received three treatments in a random order: acupuncture according to an individualised diagnosis of Chinese medicine (classical acupuncture), a semistandardised modern version of acupuncture usually employed in acupuncture trials (modern acupuncture) and non-specific needling. The main findings were a twice as large improvement in knee flexibility immediately after classical acupuncture (10.3 degrees) as compared to modern acupuncture (4.7 degrees) and no change after non-specific needling (0.3 degrees). The largest improvements in pain were also seen immediately after classical acupuncture (a WOMAC score reduction by 50% or more in 85 of 116 patients); however, non-specific needling also achieved considerable effects (core reduction by 50% in 56 of 116 patients, approaching two-thirds of the maximum effect observed after classical acupuncture. Therefore, the present data suggest substantial non-specific effects in subjective pain relief. In contrast to subjective pain relief, however, improvements in knee flexibility as objective outcome measure were *only* seen after the needling of specifically selected points and not after non-specific sham needling. To our understanding, this is the first study to prove specific effects of acupuncture and the first to exclude bias caused by differences in the control arms.

With respect to pain relief, the present study corroborates earlier findings. The measure of effect observed for the sham acupuncture as well as for the semistandardised modern acupuncture was similar to those previously observed in multicentre trials. Pain relief of comparable effect can also be achieved by other methods such as transcutaneous electrical nerve stimulation, supporting the notion that neurogenic pain contributes to the symptoms in patients with degenerative changes in joints [32, 33]. However, the non-specific effects of acupuncture may exceed those of mere placebo effects [34], for reasons as yet unexplained. 

Interestingly after seven days, no relevant difference in pain scale was reported, although we found the significant changes in knee motility to be persistent among the three treatment groups. This gain in function (knee flexibility) may be considered an indirect measure of pain relief as pain is the main limiting factor for knee motility.

Moreover, we observed a rapid improvement of knee flexibility immediately after classical acupuncture, which was twice the effect observed after modern acupuncture and absent after non-specific needling. Elucidating the physiological mechanisms [35–38] underlying this method-specific difference in effect was beyond the scope of the present study. Experimental data, however, offer some possible explanations: while the immediate effects on pain and knee flexibility exclude structural changes in the affected joints as the underlying mechanism of acupuncture in this experimental setting, they do, however, indicate an underlying neural mechanism [36]. It remains speculative as to whether this reflex-like effect involves functional changes within higher regions of the central nervous system or whether regional effects on musculoskeletal dynamics and connective tissue structures may be the dominant mechanism. The observed immediate effects, however, make a primarily systemic or humoral effect rather unlikely. As the systematic search for acupuncture points with altered perception is an integral part of history taking and work-up for the Chinese diagnosis, it is conceivable that the individualised diagnostic approach may enhance the chance to effectively identify needling points with the potential for reducing functional limitations. The present study suggests that the methodology of arriving at acupuncture points may matter. In the present study, classical acupuncture outperformed modern acupuncture. Future acupuncture studies should, therefore, consider potential differences arising from the modality of acupuncture techniques in the study design. 

### 3.2. Limitations

Several caveats of the present investigation require consideration. Firstly, we studied each acupuncture technique only once in each patient, and treatments were usually one week apart. Thus, we are unable to infer the long-term or cumulative effects of repeated applications; the study should, therefore, be considered a proof of concept study. 

The available data from the present study corroborate a rapid decline, particularly of the non-specific pain relief effect, within one week. Secondly, the present data suggest that effects on knee mobility are somewhat retained. However, the imperfect retest reliability of repeated knee-flexion measures after one week suggests viewing this result with caution and encourages repetition in other studies. Thirdly, crossover designs are prone to carry-over effects. We cannot rule out residual carry-over effects beyond those explicitly modelled within the multilevel statistical method. Finally, while the data support the notion that the choice of needling points matters, the relevant aspects of the Chinese diagnosis still remain to be elucidated. This, however, cannot be addressed in this work. 

## 4. Conclusions

In summary, our double-blinded and randomised crossover study provides a novel study design for assessing efficacy in acupuncture and establishes a framework for addressing the question as to whether the specific choice of acupuncture points matters. The study was conducted in osteoarthritis of the knee, and the outcome measures are self-reported pain relief and knee motility. As to the first, non-specific needling achieved about two-thirds of the subjective pain relief achieved after classical acupuncture, suggesting considerable non-specific effects. With respect to knee motility, individualised classical acupuncture achieved twice the effect of semistandardised modern acupuncture. No change, however, was observed after non-specific needling. This suggests a considerable specific effect of acupuncture in objective knee flexibility, an effect that appears to be method-specific as well. In the scientific discussion about efficacy of acupuncture, our data suggests that it bears both specific and non-specific effects, and the selection of acupuncture points for treatment does appear to be relevant. 

## Figures and Tables

**Table 1:** Acupuncture points chosen for nonspecific needling.

(i) A point between the gallbladder and stomach conduit at the posterior edge of the fibula 2 cun above the malleolus lateralis	
(ii) A point 2 cun and 6 cun, respectively, above the malleolus medialis on the tibial surface (intracutaneous needling without contact to the periost with the needles pointing to the knee)	
(iii) A point in the middle of the thigh on a line between the patella and the anterior iliac spine	
(iv) A point at the top of the contracted biceps muscle	
To equalise the number of needles employed between the different needling modalities, the following additional points were permitted:	
(i) A point 3 cun above and medial to the cleft of the knee joint between the spleen conduit and the renal conduit	
(ii) A point in the middle of a line between liver 13 and liver 16	
(iii) A point in the middle of a line between gallbladder 37 and vesical 58	
(iv) A point 2 cun dorsal to gallbladder 32	
(v) A point in the middle of a line between heart 2 and pericardium 3	

**Table 2:** Patient characteristics.

Characteristic	Total sample	CA as first treatment	MA as first treatment	NA as first treatment	P value
*N*	116	40	37	39	
Gender male (percent)	38 (33%)	8 (20%)	17 (46%)	13 (33%)	0.053
Age (years)	62.4	62.7	61.6	62.9	0.79
Kellgren Grade III (percent)	57 (49%)	22 (55%)	17 (46%)	18 (46%)	0.66
Left knee (percent)	54 (47%)	17 (43%)	20 (54%)	17 (44%)	0.54
Duration of pain (years)	4.7	4.9	5.3	3.9	0.50
Premedication (percent)	39 (34%)	13 (33%)	13 (35%)	13 (33%)	0.96

CA: classical acupuncture; MA: semistandardised modern acupuncture; NA: nonspecific needling. Columns describe the first treatment. All patients subsequently received the two remaining treatment modalities.

## Abbreviations

NSAID: nonsteroidal anti-inflammatory drug
NNT: number needed to treat.

## References

[1] Lawrence RC, Helmick CG, Arnett FC (1998). Estimates of the prevalence of arthritis and selected musculoskeletal disorders in the United States. *Arthritis and Rheumatism*.

[2] Felson DT, Lawrence RC, Dieppe PA (2000). Osteoarthritis: new insights. Part 1: the disease and its risk factors. *Annals of Internal Medicine*.

[3] Pendleton A, Arden N, Dougados M (2000). EULAR recommendations for the management of knee osteoarthritis: report of a task force of the Standing Committee for International Clinical Studies Including Therapeutic Trials(ESCISIT). *Annals of the Rheumatic Diseases*.

[4] Hernandez-Diaz S, Garcia-Rodriguez LA (2001). Epidemiologic assessment of the safety of conventional nonsteroidal anti-inflammatory drugs. *The American Journal of Medicine*.

[5] Bausell RB, Lee WL, Berman BM (2001). Demographic and health-related correlates of visits to complementary and alternative medical providers. *Medical Care*.

[6] Berman BM, Bausell RB, Lee WL (2002). Use and referral patterns for 22 complementary and alternative medical therapies by members of the American College of Rheumatology: results of a national survey. *Archives of Internal Medicine*.

[7] Eisenberg DM, Davis RB, Ettner SL (1998). Trends in alternative medicine use in the United States, 1990–1997: results of a follow-up national survey. *Journal of the American Medical Association*.

[8] Berman BM, Lao L, Langenberg P, Lee WL, Gilpin AMK, Hochberg MC (2004). Effectiveness of acupuncture as adjunctive therapy in osteoarthritis of the knee: a randomized, controlled trial. *Annals of Internal Medicine*.

[9] Witt C, Brinkhaus B, Jena S (2005). Acupuncture in patients with osteoarthritis of the knee: a randomised trial. *The Lancet*.

[10] Vas J, Méndez C, Perea-Milla E (2004). Acupuncture as a complementary therapy to the pharmacological treatment of osteoarthritis of the knee: randomised controlled trial. *British Medical Journal*.

[11] Scharf HP, Mansmann U, Streitberger K (2006). Acupuncture and knee osteoarthritis: a three-armed randomized trial. *Annals of Internal Medicine*.

[12] Kleinhenz J, Streitberger K, Windeler J, Gußbacher A, Mavridis G, Martin E (1999). Randomised clinical trial comparing the effects of acupuncture and a newly designed placebo needle in rotator cuff tendinitis. *Pain*.

[13] Streitberger K, Vickers A (2004). Placebo in acupuncture trials. *Pain*.

[14] Moore A, McQuay H (2005). Acupuncture: not just needles?. *The Lancet*.

[15] Boutron I, Tubach F, Giraudeau B, Ravaud P (2003). Methodological differences in clinical trials evaluating nonpharmacological and pharmacological treatments of hip and knee osteoarthritis. *Journal of the American Medical Association*.

[16] Witt CM, Brinkhaus B, Willich SN (2006). Acupuncture: clinical studies on efficacy and effectiveness in patients with chronic pain. *Bundesgesundheitsblatt Gesundheitsforschung Gesundheitsschutz*.

[17] Hummelsberger J, Ernst E, Greten HJ, Bahr F (2005). After large studies by federal health insurance: what is the future of acupuncture?. *MMW-Fortschritte der Medizin*.

[18] Kalauokalani D, Cherkin DC, Sherman KJ (2005). A comparison of physician and nonphysician acupuncture treatment for chronic low back pain. *Clinical Journal of Pain*.

[19] Zheng L, Faber K (2005). Review of the Chinese medical approach to the management of fibromyalgia. *Current Pain and Headache Reports*.

[20] Altman DG, Schulz KF, Moher D (2004). Turning a blind eye: testing the success of blinding and the CONSORT statement. *British Medical Journal*.

[21] Huwiler-Müntener K, Jüni P, Junker C, Egger M (2002). Quality of reporting of randomized trials as a measure of methodologic quality. *Journal of the American Medical Association*.

[22] Greten Kursbuch J (2003). *Traditionelle Chinesische Medizin*.

[23] Brinkhaus B, Becker-Witt C, Jena S (2003). Acupuncture randomized trials (ART) in patients with chronic low back pain and osteoarthritis of the knee: design and protocols. *Forschende Komplementarmedizin und Klassische Naturheilkunde*.

[24] Stucki G, Meier D, Stucki S (1996). Evaluation of a German version of the WOMAC (Western Ontario and McMaster Universities) osteoarthritis index. *Zeitschrift fur Rheumatologie*.

[25] Whitehouse SL, Lingard EA, Katz JN, Learmonth ID (2003). Development and testing of a reduced WOMAC function scale. *Journal of Bone and Joint Surgery B*.

[26] Brosseau L, Balmer S, Tousignant M (2001). Intra- and intertester reliability and criterion validity of the parallelogram and universal goniometers for measuring maximum active knee flexion and extension of patients with knee restrictions. *Archives of Physical Medicine and Rehabilitation*.

[27] Brosseau L, Tousignant M, Budd J (1997). Intratester and intertester reliability and criterion validity of the parallelogram and universal goniometers for active knee flexion in healthy subjects. *Physiotherapy Research International*.

[28] Enwemeka CS (1986). Radiographic verification of knee goniometry. *Scandinavian Journal of Rehabilitation Medicine*.

[29] Gogia PP, Braatz JH, Rose SJ, Norton BJ (1987). Reliability and validity of goniometric measurements at the knee. *Physical Therapy*.

[30] Goldstein H, Healy MJR, Rasbash J (1994). Multilevel time series models with applications to repeated measures data. *Statistics in Medicine*.

[31] Goldstein H, Browne W, Rasbash J (2002). Multilevel modelling of medical data. *Statistics in Medicine*.

[32] Ng MML, Leung MCP, Poon DMY (2003). The effects of electro-acupuncture and transcutaneous electrical nerve stimulation on patients with painful osteoarthritic knees: a randomized controlled trial with follow-up evaluation. *Journal of Alternative and Complementary Medicine*.

[33] Ordeberg G (2004). Characterization of joint pain in human OA. *Novartis Foundation Symposium*.

[34] Kaptchuk TJ, Stason WB, Davis RB (2006). Sham device v inert pill: randomised controlled trial of two placebo treatments. *British Medical Journal*.

[35] Yahia LH, Pigeon P, DesRosiers EA (1993). Viscoelastic properties of the human lumbodorsal fascia. *Journal of Biomedical Engineering*.

[36] Hinz B, Gabbiani G (2003). Mechanisms of force generation and transmission by myofibroblasts. *Current Opinion in Biotechnology*.

[37] Schleip R, Naylor IL, Ursu D (2006). Passive muscle stiffness may be influenced by active contractility of intramuscular connective tissue. *Medical Hypotheses*.

[38] Langevin HM, Churchill DL, Cipolla MJ (2001). Mechanical signaling through connective tissue: a mechanism for the therapeutic effect of acupuncture. *FASEB Journal*.

